# General Practitioners’ Experiences of Professional Uncertainties Emerging from the Introduction of Video Consultations in General Practice: Qualitative Study

**DOI:** 10.2196/36289

**Published:** 2022-06-14

**Authors:** Maja Nordtug, Elisabeth Assing Hvidt, Elle Christine Lüchau, Anette Grønning

**Affiliations:** 1 Department for the Study of Culture University of Southern Denmark Odense M Denmark; 2 Department of Social Work, Child Welfare and Social Policy Oslo Metropolitan University Oslo Norway; 3 Department of Public Health, Research Unit for General Practice University of Southern Denmark Odense M Denmark

**Keywords:** video consultation technology, general practice, COVID-19, doctor-patient communication, uncertainties, general practitioners, video consultation, virtual health, physician, digital health, pandemic

## Abstract

**Background:**

Uncertainties are omnipresent in health care, but little is known about general practitioners’ (GPs) professional uncertainties concerning digital consultations. This is problematic, as many countries have undergone an extensive digital transformation.

**Objective:**

The aim of this study was to explore the professional uncertainties that emerged among Danish GPs with the introduction of video consultations.

**Methods:**

We conducted qualitative interviews with 15 Danish GPs during the beginning of the COVID-19 pandemic in 2020. The interviews were analyzed using an abductive approach.

**Results:**

We identified 3 categories of uncertainty: *integrity*, *setting*, and *interaction*. Respectively, these 3 categories of uncertainty refer to (1) uncertainties related to how technology may impede the provision of health care; (2) uncertainties related to the potentials of video technology; and (3) uncertainties related to how the video consultation technology affects interactions with patients.

**Conclusions:**

The uncertainties experienced by Danish GPs appear to be a typical reaction to the introduction of new technology. Embedding video consultation technology into GPs’ working routines will take time, and GPs do not necessarily feel intuitively capable of transferring their abilities, such as being good and socially present for video-mediated consultations. The heterogeneity of professional uncertainties experienced among the GPs suggests that they are the product of individual GP-technology relationships—not of the technology in itself. Consequently, we cannot expect that uncertainties can be remedied by changing or precluding new technology.

## Introduction

### Background

“Whether a physician is defining a disease, making a diagnosis, selecting a procedure, observing outcomes, assessing probabilities, assigning preferences, or putting it all together” [1], every physician activity can be linked to uncertainty. Uncertainty in health care is a challenge many have acknowledged and made visible, and many conceptual meanings have been attributed to it. In their conceptual taxonomy of the varieties of uncertainty in health care, Han et al [2] defined uncertainty as fundamentally being “the subjective perception of ignorance” that “makes possible the many manifestations of uncertainty catalogued in conventional definitions of the term—for example, feelings of doubt, perceptions of indefiniteness, indeterminacy, unreliability, and so on.” Furthermore, uncertainties in health care vary. According to Han et al [2], the uncertainties can be disease-centered and relate to “diagnosis, prognosis, causal explanations, and treatment recommendation”; they can relate to “structures and processes of care”; or they can be personal and relate to “psychosocial and existential issues.”

One aspect of increasing pertinence for health care is assessing the impact of the digital transformation of health care services, such as the implementation of digital consultations, including both email and, most recently, video consultations. Although video consultations are not “straightforward replacements” of physical consultations [3], they are increasingly being used for certain health problems [4]. The important insights about uncertainties in health care mentioned above have not yet been translated to health care professionals’ uncertainties related to digital consultations. Consequently, little is known about the role that technologies play in health care professionals’ experiences of uncertainty, and even less is known about how feelings of uncertainty concerning digital consultations can be reduced to optimize care and health care professionals’ well-being. This is particularly problematic considering how modern health care systems in many countries have undergone a digital transformation in the past decades, which was accelerated during the COVID-19 pandemic. Research shows that technology can either alleviate clinicians’ stress or contribute to it, depending on its perceived usability [5,6].

During the COVID-19 pandemic in Denmark, video consultation technology was rapidly implemented in general practice and made available to all patients via the mobile app *Min Læge* (“My Doctor” in Danish) as an alternative to face-to-face consultations. Moreover, Danish health authorities encouraged patients to use video consultations to decrease the transmission of COVID-19 [7,8]. From the beginning of the pandemic in March 2020 to December 2020, 2.6% of all general practice consultations in Denmark were video consultations (not including email and telephone consultations) [9]. The number of video consultations peaked during the first lockdown period in March. However, as society gradually reopened, a rapid decline in video consultations occurred [10]. Although a collective agreement between the Organisation of General Practitioners and Danish Regions suggests and financially supports the continuous use of video consultations in general practice [11], 2.6% suggests a limited adaptation to this new consultation form that might be related to perceived uncertainties among general practitioners (GPs). Research shows that successful adoption of technology depends on continuing use [12]. However, as put forward by Carey and Martin [12], “[un]certainty is a core characteristic associated with new media,” and in organizations, employees may not accept the implementation of a new media service even if the organization in question needs it. The introduction and implementation of technology for new patient-provider communication practices, such as video consultations, may also be associated with uncertainties related to new expectations, roles, and working procedures [13-16]. In relation to the recent implementation of video consultations, GPs were found to have mixed experiences, and there is some ambivalence regarding the benefits. Even though some GPs find video consultations to be advantageous, for instance, in that they invite concise and focused consultations, others have indicated disadvantages, such as losing a sense of connection and intimacy with their patients [17]. Furthermore, due to concerns about missing patients’ symptoms, some GPs feel that video consultations can compromise the quality of medical work [18].

### Objective

To date, few studies have been conducted on GPs’ perceived uncertainties related to video consultations, which were rapidly implemented due to the COVID-19 pandemic [19]. With this study, we sought to explore this gap in the research. More specifically, we asked the following question: which professional uncertainties among Danish GPs emerged with the introduction of video consultations in connection with COVID-19?

Exploring this question will provide insights into how the sustained use of video consultations in general practice in Denmark and elsewhere can be optimized, as identifying and categorizing uncertainties can help to address them.

## Methods

### Data Collection

This study is part of a larger qualitative research project exploring the implications of the use of video consultations as experienced by Danish GPs and patients. The data corpus in the project consists of semistructured interviews with users of video consultations (patients and GPs) and recordings of video consultations. In this paper, we draw on data from interviews with 15 GPs from different clinics: 8 men and 7 women aged 39-61 years from 4 (out of 5) different Danish regions (see Table 1). Of the 15 GPs, 9 had not used video consultations before the onset of the pandemic. Of the GPs who had conducted consultations through video before the pandemic, 5 had used it as persons testing the video technology system, of which 4 had only used it to a very limited extent. The GPs we interviewed were not selected based on any specific inclusion or exclusion criteria, but rather through a convenience sampling technique [20]. This is due to not many GPs having experience with video consultations at the time of our data collection. We were, however, able to secure a variation in the GPs’ age and gender and in which region they worked.

Semistructured interviews with the GPs were conducted by ECL and a junior researcher either by phone, video (Zoom, Teams), or at a physical meeting in the clinic. The interviews followed a semistructured interview guide and took place in 2020 during the beginning of the COVID-19 pandemic. The purpose of using a semistructured interview guide was to understand video consultations from the perspective of the GPs [21], allowing room for individual opinions and experiences. Adjustments were made to the interview guide during the period of data collection, as interviewees inspired new interview questions. Examples of interview questions include the following: “How have you experienced participating in video consultations with patients?”; “What does it mean for your professional relationship with the patient that your consultation takes place via a screen?”; and “Did you feel comfortable letting your consultation take place as a video consultation? If yes/no, what was the reason?” Interviews were audio-recorded and transcribed verbatim by ECL and a student assistant concurrent with the data collection.

**Table 1:** General practitioner characteristics.

Variable	Our participant sample
Age	39-61 years
Gender	8 men and 7 women
Geographical dispersion	4 out of 5 Danish regions

### Data Analysis

The analysis was conducted and developed by the authors of this paper: 3 with a background in media studies and 1 with a background in humanistic health research. All authors have an academic interest in health and media, and 3 have general practice as their clinical research setting and collaborate with GPs who are also researchers. All authors carefully read the transcribed interviews. For the purpose of this paper, we analyzed the data through 2 major stages of coding: first stage and second stage. In the first cycle of coding, researchers initially assign codes to data chunks; in the second cycle of coding, researchers then work with these codes by grouping the codes, such as into categories or themes [22]. MN initially read the interviews and identified various uncertainties through inductive coding using the computer-assisted qualitative data analysis software NVivo (version 12; QSR International). This process served as the first cycle coding of the data [22]. The identified uncertainties were discussed among all authors, and in the second cycle of coding [22], we grouped the data from the coding into 3 general categories. Previous research on uncertainties [2,23] informed our discussions of the data material and our abductive analysis [24]. As such, we went back and forth between the analytical work and previous research, allowing for new insights to emerge from the data while also considering existing research [25]. Through this abductive process, we were able to contribute to the existing research on uncertainties while also not being constrained by previous understandings and classifications. Therefore, we were able to contribute new insights that were built upon previous knowledge.

In our analysis, we focused on GPs’ uncertainties in relation to video consultations by focusing on the experiential aspect, as experience “is the place where the mutual relation between human beings and their world can be localized” [26].

### Ethics Approval

All patients gave written consent and were informed that participation in the study was voluntary. The study was approved by the institutional board of the University of Southern Denmark and the Research and Innovation Organisation (approval number 10.971) and was conducted in accordance with the General Data Protection Regulation.

## Results

### Analytical Categories

Below, we present the analytical categories *integrity*, *setting*, and *interaction* using examples that show the nuances and heterogeneities in the uncertainties that the GPs experienced in connection with the introduction of video consultations. The quotes used to exemplify were translated from Danish to English. Each category includes 2 or 3 subcategories, as shown in Table 2.

The categories are not sharply delimited. Rather, they are fluid categories that can help with understanding the variety of the GPs’ uncertainties; hence, they are not mutually exclusive. This also means that the categories partially overlap.

**Table 2:** Categories of professional uncertainties that emerged among Danish general practitioners with the introduction of video consultations.

Category	Subcategory
Integrity	medical diagnostic ethical responsibilities juridical responsibilities
Setting	off-screen setting on-screen setting
Interaction	challenges with focusing and concentrating challenges with staying present in the web-based environment challenges with showing emotions in the mediated environment without being able to physically touch each other

### Integrity

The category *integrity* comprises the uncertainties that the GPs experienced when having to execute their medical responsibilities through a video consultation. We understand *integrity* in this study as something that “creates the ethical obligation to adhere to standards of intellectual and moral excellence in individual patient care” [27]. The uncertainties relating to integrity revolve around how the use of video consultation technology might impede the provision of health care, potentially bringing the GPs’ professionalism into disrepute. Integrity-related uncertainties include the following subcategories: (1) medical diagnostic, (2) ethical responsibilities, and (3) juridical responsibilities.

First, the GPs experienced uncertainties related to medical diagnostics. The GPs expressed how they wished to provide their patients with high-quality care but felt their level of service was compromised due to the use of video consultations. Consequently, some of the GPs expressed that video consultations made them feel “so far away from the GP I want to be” (GP #2). Furthermore, one GP explained, “Just because video has been introduced, we shouldn’t create a discounted version of general practice” (GP #3).

In general, the uncertainties relating to medical diagnostics revolved around the GPs feeling sensorily limited due to the video consultation technology only offering the GP a partial view of the patient and precluding the GPs’ other senses, including the ability to smell, touch, and feel. Thus, the video consultation technology limited the information available about the patient. The GPs referenced how they would normally get information about the patient “for free” in physical consultations where all their senses could be used. Specifically, the inability to use all senses was associated with situations in which information was retrieved that exceeded the actual medical problem the patients had made an appointment for. One GP explained the following:

I can’t see how they get up from the waiting room and how they enter the consultation room. I don’t see how they huff and puff, because when I talk to them in the video consultation, they are sitting still in their chair.

GP #5

Similarly, another GP explained that “sometimes, you also use your sense of smell to see if people smell of alcohol or are well-groomed, or what do I know” (GP #8).

As such, through video consultations, GPs explained that they do not have the same fine-tuned way of sensing their patients as they do in physical consultations. Moreover, some of the GPs expressed uncertainties in connection with the assessment of birthmarks, details of skin eruption, colors, weight, and sore throats through video consultations. These uncertainties appeared to be ascribable to a fear of providing a poorer medical service and inefficient care when consultations were conducted over video. As one GP said, “if you increasingly introduce video consultations in general practice, then you compromise your medical professionalism and you compromise the efficiency” (GP #5).

Second, uncertainties related to integrity also included ethical responsibilities. The GPs came to question their own ethical behavior as a result of having their senses limited. For instance, one GP questioned the ethical appropriateness of asking a patient to film body parts (eg, their thighs and behind) to gather more information about the patient, such as if a patient was suspected to have put on weight: “Well, we might get better at [asking], ‘Could you film all of you?’ Or…But it’s also a little boundary-crossing, you know?” (GP #2).

Similarly, another GP questioned whether patients perceived it as intimidating to get undressed in front of a video camera. The GP described their considerations as follows:

It might be that it is easier for them to show—how do you put it?—eczema on their breast, and that it’s me who finds it more intimidating that they have to sit in their [own] bathroom and lift up their breast, you know?...They [might] think it’s nicer there, than sitting in some man’s office taking off their bra.

GP #9

Third, uncertainties related to integrity also included whether GPs can legally stand by the assessments they make over video. Not many GPs explicitly expressed these uncertainties, but one GP stated the following:

You’re behind on points. If something goes wrong...then, naturally, there’s a big difference in whether you have seen the patient or not seen the patient from a medico-legal point of view.

GP #1

The GP further expressed legal uncertainties about whether seeing a patient through video could be acknowledged as “a proper basis for diagnosing and starting treatments” (GP #1). Consequently, this GP expressed uncertainties about whether the use of video consultations could create legal issues, potentially challenging or risking their professionalism.

Above, we described the 3 subcategories of uncertainties related to integrity. We identified integrity uncertainties among most of the GPs we interviewed. Some, however, expressed that they were not affected by uncertainties related to integrity, as can be seen in the following answer to the question on whether the GP felt professionally safe when diagnosing a patient through a screen: “Yes, I felt comfortable with that” (GP #9). Similarly, another GP answered an interview question on whether they felt sufficiently professional when seeing a patient over video by saying “Yes, I haven’t really had any concerns about that” (GP #7).

Some GPs were thus comfortable with making decisions about patients’ health after having seen them through a video consultation. Although recognizing that some aspects of patients’ health issues might be missed in a video consultation (one GP, for instance, expressed when using video consultations, they missed the last 5% of the total amount of information available about the patient), it was highlighted that “[GPs] could get better at making decisions without [the last 5%]” (GP #12). Thus, the GP suggested that it could be possible to make decisions about a patient’s health even if GPs are missing the small percentage of information that the video consultation does not transfer regarding the patient.

### Setting

The category *setting* comprises the uncertainties that the GPs experienced in relation to the video technology’s potentials. This category relates to the execution of video consultations and includes the following subcategories: (1) off-screen setting and (2) on-screen setting.

First, uncertainties related to off-screen setting emerged because, for many GPs, the video consultation had not become a routine consultation form. Rather, most of the GPs were thrown into the use of video consultations because of the pandemic. Consequently, routines and habits related to video consultations did not exist for patients, GPs, or other health professionals in the GP clinic. One GP questioned how to build a video consultation culture among patients and explained how the patient “also has to sit down, also has to sit in a room without interferences. They can’t be on their way to the bakery, they can’t be driving their car or...” (GP #5). The GP added that “my own culture around having a video consultation is not very developed” (GP #5).

Another GP explained how hybrid practices (ie, the alternation between onsite and video consultations) were not part of everyday life in the clinic and at times created awkward situations:

...it’s a bit awkward when I’m sitting and looking in [to the computer], and then a resident comes knocking on my door and “knock knock knock” and “Could you help me with a blood sample?”

GP #9

Thus, the GPs were uncertain about how to deal with the off-screen aspects of video consultations.

Uncertainties related to the category *setting* also included on-screen setting uncertainties. Some GPs experienced difficulties with using the computer software when consulting. For example, they considered the new procedure for booking video consultations difficult and found it tricky to look at files on the computer while also having the image of the patient on the screen. Furthermore, some GPs feared that they would unintentionally share their screen: “I might accidentally share some things that I shouldn’t share with them [during the video consultation]” (GP #5).

Related to the execution of the consultation, we also identified uncertainties among the interviewed GPs regarding how to adjust their consultation practice to the video medium. One GP talked about their uncertainties regarding how to look at a patient’s journal on the computer that was also being used for the video consultation.

And the patients, for instance, can’t see that as of right now I’m looking in their journal. They can’t follow in the same way as if they are sitting here [at the GP’s office], then they can see that right now I’m looking at blood samples from the last time [they visited]. If they’re sitting at home, they can only see my head and I have to say out loud: “You know what, just a moment. I’ll go look,” and then you have to remove the image and such.

GP #9

In sum, the lack of established culture and practices around video consultations created uncertainties about how to create the best setting for a video consultation.

### Interaction

The category *interaction* comprises the uncertainties that emerged in the GPs’ interactions with patients in video consultations. Examples of these uncertainties are presented in the following 3 subcategories: (1) challenges with focusing and concentrating, (2) challenges with staying present with the patient in the web-based environment, and (3) challenges with showing emotions in the mediated environment without being able to physically touch each other.

First, challenges with being able to focus and concentrate, especially during longer consultations (eg, talk therapy), were mentioned by several GPs. They stated that they experienced uncertainties related to staying focused and the risk of turning to other tasks during video consultations, such as reading emails. One GP explained the following:

And then I also just think it’s difficult to concentrate. Basically, you can do all sorts of things at the same time, read emails and reply to things.

GP #2

Moreover, the ability to focus and concentrate was experienced as challenging because computers are used for other tasks, such as communication on social media and messages of various kinds (Messenger, Facebook). For example, notifications from other programs that popped up behind the video consultation were perceived as distracting, or as one GP explained: “When you’re sitting with your main communication organ in front of you in which all your Facebook thingies appear, then that is constantly disrupting in the background” (GP #4).

Second, regarding the uncertainties connected to the inability to stay present with a patient in the web-based environment, the above-cited GP emphasized that they felt they were not fully present in the video consultation, stating simply, “I’m not as present, I mean” (GP #2).

Furthermore, some, though not all, GPs experienced difficulties with making eye contact with their patients, to which relational importance was accorded. One GP said the following:

But I think it’s hard for me to have eye contact with the patient. I have to have eye contact with the camera, but I can’t see if they...I mean, I have to choose: should they see my eyes, or should I see theirs, you know?

GP #4

Using video consultations may cause GPs to feel as if they must choose between looking at their patient and allowing the patient to feel as if they are making eye contact with their GP. However, when asking the GPs whether they experienced the ability to establish and maintain eye contact throughout a video consultation, some GPs answered affirmatively: “Yes, I actually do” (GP #8).

Third, especially when it came to consultations dealing with emotional issues, several GPs experienced uncertainties due to the perceived challenge of showing emotion when being unable to be near or physically touch the patient. For example, one GP stated that “It’s difficult to have someone crying or in shock on the screen. I think the physical—there’s a kind of contact [in that situation] that on screen becomes sort of cold and cynical” (GP #15).

Several GPs mentioned that they perceived a lack of potential for action to compensate for the fact that they could not touch the patient. Moreover, one GP also mentioned uncertainties regarding ascertaining a patient’s emotional state during a video consultation, questioning whether a patient was about to start crying or just staying silent for a short while. However, the same GP summarized that they found that they had managed this part of video consultations well, mentioning that perhaps this was due to the relational continuity that they were holding with the patient.

## Discussion

### Principal Findings

Through our analysis, we have contributed new insights on uncertainties in health care by exploring technologically mediated uncertainties as perceived by GPs in Denmark in the period right after the rapid implementation of video consultations in general practice [2,23]. Little has been known about health care professionals’ experiences of uncertainty in relation to technology; our study shows some of the challenges GPs experience when new technology is introduced into their working life.

We have identified 3 categories through which we can understand how video consultations create professional uncertainties among some Danish GPs: *integrity*, *setting*, and *interaction*. These categories are closely connected to and mutually shape one another.

In our analysis, the video consultation technology came to play a substantial role in shaping the GPs’ experience of their professional uncertainties as having been compromised. The uncertainties related to *integrity* show that the GPs were concerned about whether the video consultation technology would impede the provision of care and, consequently, whether the technology could challenge the GPs’ ability to provide quality in health care and thus be the GPs they wanted to be. However, uncertainties are omnipresent in health care, and, as mentioned in the introduction, the implementation of new media is characterized by uncertainty [12]. Historically, the introduction of new communication technologies has led to concerns among GPs. For instance, when the telephone was introduced in general practice, GPs were concerned about the potential extra workload it might create [17,28]. Furthermore, when email consultations were introduced, GPs were found to have concerns about workload, safety, inappropriate use, and confidentiality, as well as difficulties related to diagnosis [17,29]. Consequently, GPs experiencing uncertainties upon the introduction of video consultation technology appears to be a typical reaction.

Technologies are not automatically accepted; they must go through a process until they become normalized, pointing further to the uncertainties related to *setting*. GPs were found to have uncertainties relating to both off-screen and on-screen settings, and the analysis suggests that the practice of video consultation was not yet routinely embedded in general practice [30]—the use of video consultations has not become normalized and well-integrated into the context of general practice consultations. The processes through which a practice becomes routine are complex [30], and when introducing new technology in a profession such as general practice—a profession characterized by rigor and structure [31]—it is to be expected that the implementation process will be complex as well. Thus, adjustment time for GPs to embed the technology into their everyday working life is needed.

For video consultation technology to become routinely embedded in general practice, in addition to not feeling that the technology impedes the provision of health care, GPs must also feel that the interaction through the medium is satisfactory. This points to the uncertainties related to *interaction*. These uncertainties show that some GPs experienced challenges with focusing and concentrating, staying present, and showing emotions. This can be related to the ongoing scholarly discussion about uncertainties that emerge in the digital sphere in general and concerns about the implementation of new technology (telephone, email, and video) in particular. Uncertainty in the digital sphere has been studied for almost 5 decades, focusing on the question of how one perceives other people during a computer-mediated interaction, also termed “social presence” [32,33] and “closeness” [34,35] and related to degrees of “intimacy” [36,37]. The question of to what extent it is possible to perceive presence and closeness through a screen often leads to subjective answers, ranging from distancing to super-connectedness. Hence, an essential point is that users can perceive the same technology’s potential for presence and closeness in vastly different ways.

Our analysis also shows that the perceptions and experiences of uncertainties vary, suggesting that uncertainties are not entirely a product of the technology but rather of the GP-technology relationship—a point emphasized at large in science and technology studies [26]. Consequently, different GP technologies are the sources of different uncertainties as reflected in the analysis; for instance, some GPs were comfortable diagnosing patients using video technology, whereas others feared that the quality of their work would be impeded. Similarly, some GPs experienced difficulties with making eye contact with their patients, whereas others did not.

Looking at the above identified GP uncertainties as relationally grounded, the implementation of video consultation in general practice must be acknowledged as “not exclusively a technological issue, but part of a complex organizational change” [38], involving the processes of relating, adapting, or resisting among the whole clinic personnel. In this light, uncertainties cannot necessarily be remedied by changing or precluding the technology but rather by considering the relation between clinic users and the technology when implementing new consultation forms. To ease GPs’ uncertainties, future research could delve further into the relations between GPs and technology; the more we know about the relations between GPs and technology, the more we can act on and limit their uncertainties by way of education that may increase feelings of confidence and self-efficacy. However, we also acknowledge that some of the uncertainties experienced by GPs can be a legitimate reaction to an inappropriate use. Video consultations are not suitable for all health issues, and furthermore, whether a health issue is suitable for video consultation may also be dependent on the individual competences and experiences of the GP and the patient.

### Limitations

This study was conducted during an early stage of the introduction of video consultations in general practice; therefore, it cannot state anything about its adaptation over time. Furthermore, as this is a qualitative study, we are unable to make claims about the general frequency of the uncertainties of GPs in Denmark. However, we have no reason to believe that the sample of GPs in this study is not transferrable to other GPs in Denmark.

## Abbreviations

GP: general practitioner
